# Getting Into the Brain: The Intranasal Approach to Enhance the Delivery of Nerve Growth Factor and Its Painless Derivative in Alzheimer’s Disease and Down Syndrome

**DOI:** 10.3389/fnins.2022.773347

**Published:** 2022-03-09

**Authors:** Simona Capsoni, Antonino Cattaneo

**Affiliations:** ^1^Bio@SNS Laboratory of Biology, Scuola Normale Superiore, Pisa, Italy; ^2^Section of Physiology, Department of Neuroscience and Rehabilitation, University of Ferrara, Ferrara, Italy; ^3^European Brain Research Institute–Fondazione Rita Levi-Montalcini, Rome, Italy

**Keywords:** nerve growth factor, down syndrome, intranasal, neurogenesis, inflammation, microglia, astrocytes

## Abstract

The neurotrophin Nerve Growth Factor (NGF) holds a great potential as a therapeutic candidate for the treatment of neurological diseases. However, its safe and effective delivery to the brain is limited by the fact that NGF needs to be selectively targeted to the brain, to avoid severe side effects such as pain and to bypass the blood brain barrier. In this perspective, we will summarize the different approaches that have been used, or are currently applied, to deliver NGF to the brain, during preclinical and clinical trials to develop NGF as a therapeutic drug for Alzheimer’s disease. We will focus on the intranasal delivery of NGF, an approach that is used to deliver proteins to the brain in a non-invasive, safe, and effective manner minimizing systemic exposure. We will also describe the main experimental facts related to the effective intranasal delivery of a mutant form of NGF [painless NGF, human nerve growth factor painless (hNGFp)] in mouse models of Alzheimer’s disease and compare it to other ways to deliver NGF to the brain. We will also report new data on the application of intranasal delivery of hNGFp in Down Syndrome mouse model. These new data extend the therapeutic potential of hNGFp for the treatment of the dementia that is progressively associated to Down Syndrome. In conclusion, we will show how this approach can be a promising strategy and a potential solution for other unmet medical needs of safely and effectively delivering this neuroprotective neurotrophin to the brain.

## Introduction

The neurotrophin Nerve Growth Factor (NGF) (Levi-Montalcini, 1952) has been suggested to play a neuroprotective factor in several neurological diseases and has been a matter of numerous basic and clinical research studies.

Nerve growth factor is produced from a gene located on chromosome 1 (Francke et al., 1983) as a precursor proNGF which exists in two distinct isoforms of 27 and 35 kDa (Edwards et al., 1988). ProNGF is then cleaved to mature NGF by a group of enzymes including furin and metalloproteases (Cuello et al., 2019). Recently, it has been found that both proNGF and mature NGF can trigger biological responses (Lee et al., 2001), most often with an opposite sign. First, it has been demonstrated that proNGF is the major species of this neurotrophin both in the Central Nervous Systems (Fahnestock and Shekari, 2019). Secondly, proNGF can bind p75^NTR^, the receptor common to all neurotrophins. But it can also bind to the tropomyosin-related kinase receptor TrkA, although with a lower affinity than mature NGF (Fahnestock et al., 2004). Thus, a subtle balance between the amount of proNGF, mature NGF and their receptors can lead to either a neuroprotective or a pro-neurodegenerative outcome (see below).

## Nerve Growth Factor in Alzheimer’s Disease and its Limits to Clinical Application

Alzheimer’s disease (AD) neuropathology is distinguished by deposits of misfolded proteins, mainly consisting of hyperphosphorylated tau and β-amyloid (Aβ) (Selkoe, 2001). Another prominent feature of the neurodegenerative process characterizing AD is the occurrence of cholinergic deficit (Whitehouse et al., 1982), which put the theoretical basis of the pharmacological therapy available for AD patients (use of cholinesterase inhibitors) (Giacobini, 2006). Basal forebrain cholinergic neurons (BFCNs) were identified as the most significant NGF-sensitive population inside the CNS. These neurons express both NGF receptors TrkA and p75^NTR^ (Hefti, 1986; Holtzman et al., 1992), are able to retrogradely transport NGF from their cortical projections up to their cell bodies (Seiler and Schwab, 1984) and respond to administration of exogenous NGF, in terms of increase of cholinergic phenotypical markers (Gnahn et al., 1983; Mobley et al., 1986). Most importantly, NGF is able to prevent BFCN death or atrophy, following axotomy (Hefti, 1986; Williams et al., 1986; Kromer, 1987) or linked to aging (Fischer et al., 1987). In AD, a selective decrease in the expression of TrkA, and not p75^NTR^, occurs in BFNCs and hippocampus and it correlates with the severity of the disease (Mufson et al., 2019). The distinctive cholinergic deficit in AD, together with the BFCN being NGF target neurons, has led to propose the use of NGF as a treatment for AD (Tuszynski et al., 2005; Mitra et al., 2019).

Work with the anti-NGF AD11 mouse model (Ruberti et al., 2000), in which the expression of antibodies against mature NGF in the adult brain causes a progressive neurodegeneration which is similar to that observed in AD brains, provided the first demonstration that deficits in NGF signaling may lead to a Alzheimer-like neurodegeneration (Capsoni et al., 2011), which is broader than a pure cholinergic deficit. This comprehensive neurodegeneration phenotype suggested that other cells in the brain, in addition to BFCNs, might respond to NGF deficits and, conversely, might represent targets for NGF therapeutic actions. Indeed, triggered by this neurodegeneration picture, we found that microglia are NGF target cells and respond to NGF by activating a potent and broad neuroprotective and anti-inflammatory action (Rizzi et al., 2018).

Deficits in NGF processing or transport could be causally linked to the onset of AD neurodegeneration. Whilst in the AD11 model the NGF deficit is determined by interference with an anti-NGF antibody expressed in the brain, different pathological mechanisms could result in a reduced NGF bioactivity. Thus, a reduced NGF bioactivity might result either by a defect in NGF retrograde transport system (Mufson et al., 1995) or by an unbalance of proNGF vs. mature NGF signaling (Fahnestock et al., 2001; Podlesniy et al., 2006). Indeed, experimentally increasing proNGF in transgenic mice also induces a progressive neurodegeneration, despite concomitant higher-than-normal mature NGF levels (Tiveron et al., 2013; Fasulo et al., 2017).

We can therefore formulate an NGF hypothesis for AD neurodegeneration, whereby a common link behind AD neurodegeneration is a failure or an insufficient NGF signaling, leading to inadequate neurotrophic support (Capsoni and Cattaneo, 2006; Cattaneo et al., 2008; Cattaneo and Calissano, 2012). The failure or unbalance of NGF support could be due to different causes in the overall cascade(s) of events involving NGF bioactivity: (1) decreased NGF synthesis, (2) unbalanced or altered processing, (3) alterations in receptor expression and/or activity or expression ratios, and (4) altered retrograde transport. These events would be “located” upstream of the “amyloid cascade,” which is the central core of AD neurodegeneration, as currently described (Selkoe, 2000), and would be part of a negative feedback loop that involves several steps (e.g., links between APP, tau, and axonal transport). On the other hand, the intrahippocampal injection of Aβ oligomers in naïve rats is sufficient to induce a proNGF/NGF unbalance (Bruno et al., 2009).

Thus, an initial deficit in NGF signaling or processing or a reduction of TrkA receptors will result in a feed-forward pathological cycle leading to increased accumulation of Aβ and propagation of proNGF/NGF homeostasis deficits (Cattaneo and Capsoni, 2019).

Within this theoretical frame, any therapy aimed at re-establishing the correct balance between ligands (and receptors) of the NGF pathway appears to have a clear rationale. The most direct therapeutic approach along these lines would be, therefore, to exploit NGF itself. However, the viable clinical application of NGF requires providing a solution to major obstacles, namely finding a more effective NGF delivery to the CNS and limiting adverse effects deriving from undesired NGF actions, most notably, pain.

## Past and Ongoing Nerve Growth Factor Clinical Trials in Alzheimer’s Disease

One approach to overcome the limits of NGF administration might be the use of small molecules that could cross the blood brain barrier and mimic NGF action, improving survival of target cells [Pediaditakis et al. (2016a,b) and reviewed in Gascon et al. (2021)]. Currently one of these small-molecule NGF mimetics, the P75^NTR^ binding molecule LM11A-31, is under evaluation in clinical trials in Alzheimer’s disease (Yang et al., 2008, 2020). However, this approach might represent limitations due to a more restricted pharmacological profile with respect to that of the NGF protein.

To achieve a therapeutic concentration of NGF in the brain, while also avoiding systemic exposure, a first clinical has been performed in which an intracerebroventricular infusion was performed in three patients (Eriksdotter Jonhagen et al., 1998). Despite an increase in nicotinic receptor expression and an amelioration in cognitive function, the trial had to be stopped due to the onset of unbearable back pain linked to the diffusion of NGF in the CSF irrorating the spinal cord. For this reason, subsequent clinical trials were performed using cells engineered to secrete NGF or adenoviruses carrying the sequence encoding for NGF, stereotaxically implanted by neurosurgery close to the basal forebrain. In 2005 a clinical trial targeting the BFCNs was performed in 8 patients in which autologous fibroblasts were engineered to produce NGF. Using this approach, slowing down of the cognitive decline, associated with an amelioration of cortical glucose uptake, was found (Tuszynski et al., 2005, 2015). Lately, an NGF-encoding adeno-associated viral vector also injected in the basal forebrain has been used (Rafii et al., 2014) but the treatment did not lead to clinical efficacy, most likely because of the failure to accurately engage the target cells (Rafii et al., 2018).

More recently, clinical trials using the encapsulated cell biodelivery (ECB) have been started. The ECB cells engineered to secrete NGF are located at the tip of a catheter formed by a semipermeable membrane to allow the exchange of NGF and nutrients in the extracellular fluid (Lindvall and Wahlberg, 2008). These catheters have been implanted in the basal forebrain of AD patients and allow to achieve an increase in choline acetyltransferase activity and glucose content in the brain, and amelioration in memory tests (Mitra et al., 2019). However, despite the encouraging results, the trials have been slowed down because of the variability of results due to degeneration of the engineered cells (Mitra et al., 2019).

## The Advantage of Intranasal Delivery vs. Ocular Delivery

To bypass the blood brain barrier, intranasal delivery is an alternative solution that has been proposed for several proteins (Dhuria et al., 2010; Malerba et al., 2011). As far as NGF is concerned, in 1997 Frey’s group used radioactive labeled NGF to demonstrate that the intranasal delivery allows to obtain NGF in therapeutic concentrations in several regions of rat brain (Frey et al., 1997). Several hypotheses have been formulated concerning the pathways through which the protein can reach the brain. These include nerves (olfactory and trigeminal) connecting the nasal passages to the brain, vasculature, cerebrospinal fluid (CSF) and lymphatic system [reviewed in Dhuria et al. (2010) and Malerba et al. (2011)]. Our laboratory first applied this technique in anti- NGF AD11 mice, and we showed that intranasally delivered NGF could reduce memory deficits and the accumulation of Aβ deposits, hyperphosphorylated tau and cholinergic deficiency (Capsoni et al., 2002; De Rosa et al., 2005). In a subsequent study, intranasal delivery of NGF was compared to the administration of NGF eye-drops. It was found that the ocular delivery of NGF was less efficient than nasal delivery in rescuing tau-related neurodegeneration in AD11 mice, since a ten times higher dose than the one used for intranasal delivery was necessary to obtain the same effect (Capsoni et al., 2009).

## Microglia as a New Target for the Actions of Intranasal Painless Nerve Growth Factor (Human Nerve Growth Factor Painless)

Intranasal delivery allows not only to reach brain regions, but it also reduces the possibility to have a systemic leakage of the protein in blood circulation, thus reducing the possibility to trigger side effects such as pain. To increase the therapeutic index and to reduce the possibility to trigger nociceptor sensitization, a mutation in the human NGF gene, inspired by a rare human disease, the Hereditary Sensory and Autonomic Neuropathy type V (HSAN V), was introduced. HSAN V patients carry a mutation from arginine 100 to tryptophan (R100W) and suffer of pain insensitivity without having cognitive deficits (Einarsdottir et al., 2004). After screening different amino acid substitutions, we selected the mutation R100E because of (i) its similarity to the R100W mutation in selectively altering TrkA signaling, (ii) in abolishing the binding to p75^NTR^ receptor and because of (iii) a more efficient production in *Escherichia coli* (Covaceuszach et al., 2010). In addition to the R100E mutation, a second one (P61S) was introduced to make the protein detectable against the endogenous human NGF (Covaceuszach et al., 2009). The mutant NGFP61SR100E [painless NGF or human nerve growth factor painless (hNGFp)] was shown to have the same neurotrophic potency as wildtype NGF, in several bioassays, while showing a greatly reduced pain sensitization potency, in a number of pain assays, with respect to wild type NGF (Malerba et al., 2015). From the pharmacological point of view, hNGFp is a TrkA-biased agonist, with a greatly reduced ability to bind and activate p75^NTR^ (Cattaneo and Capsoni, 2019).

In a first study, hNGFp was used to treat AD11 and APPxPS1 mice (Capsoni et al., 2012). We showed that the intranasal delivery was able to improve memory in both transgenic models, as assessed by novel object recognition and in Morris water maze tests. Moreover, in both mouse models Aβ deposition was lowered in both transgenic mice. In AD11 mice, also tau hyperphosphorylation and cholinergic deficit were decreased.

A second paper in which the treatment was performed in 5xFAD mice allowed us to uncover the neuroprotective mechanisms through which hNGFp acts to reduce the neurodegeneration and to compare the effectiveness of intranasal delivery vs. a local delivery to cholinergic neurons, mimicking the approach used in clinical trials (Capsoni et al., 2017). First, we demonstrated that intranasal hNGFp can be detected at 6 and 24 h after the administration in the hippocampus and cerebral cortex, respectively, two areas highly affected by the neurodegeneration. We found that the local delivery of hNGFp to cholinergic neurons of the nucleus basalis was not decreasing the number of plaques in 5xFAD mice, despite the sprouting of cholinergic fibers. On the contrary, with the intranasal delivery we obtained a reduction in the plaque load because of a reduced pro-amyloidogenic processing of APP and a clearance of deposited Aβ by microglia. Indeed, we found that microglia are the first cellular target of hNGFp, being the only cellular type, beside BFCNs, which express TrkA in 5xFAD mice. Thus, mechanisms through which intranasal hNGFp affects APP processing does not go through BFCNs but involve a modulation of fine cytokines, including Interleukin1α and CXCL12 which we demonstrated to be upregulated in neurons after hNGFp administration as a consequence of the blockade of Tumor Necrosis Factor α (TNFα) by its soluble receptor type 2 (Capsoni et al., 2017). The data on phagocytosis of Aβ oligomers were confirmed in a parallel study performed on primary microglia cells in which it was demonstrated that NGF can increase their micropinocytosis, thus preventing the decrease in neuronal spines and the onset of deficit in long term potentiation (LTP) (Rizzi et al., 2018). LTP was also improved in the entorhinal cortex of 5xFAD mice after intranasal treatment and this correlates also with an amelioration of memory deficits (Capsoni et al., 2017).

In conclusion, a therapeutic effect able to prevent or clear Aβ deposition in the brain of the 5xFAD mouse model required a broad hNGFp biodistribution, such that could be achieved by the intranasal delivery of hNGFp, but not by the local delivery to the basal forebrain. Thus, the intranasal, but not the local, delivery of hNGFp appears to be necessary to permeate the brain with hNGFp, reach microglia which are widely distributed in the brain and provide neuroprotection and anti-neurodegenerative effects (Rizzi et al., 2018).

## Efficacy of Intranasal hNGFp in Down Syndrome Mice

The fact that microglia is a target cell of NGF in the brain (Rizzi et al., 2018) and that is a primary target of intranasal hNGFp in the 5xFAD Alzheimer’s model (Capsoni et al., 2017) suggests that the microglia-mediated broad neuroprotective actions of hNGFp might be exploited in other disease states, in addition to AD. We tested this hypothesis by investigating the efficacy of hNGFp in a mouse model of Down Syndrome (DS). A progressive dementia is a common age-related clinical aspect of DS patients (Hartley et al., 2015), the neurodegeneration including the deposition of β amyloid, neurofibrillary tangles and cholinergic deficit in BFCNs (Hefti, 1986). Abnormal levels of the amyloid precursor protein APP found in Ts65Dn mice (Choi et al., 2009) lead to an impaired transport of NGF to BFCNs (Salehi et al., 2006) and the local infusion of NGF rescues the cholinergic deficit in these mice (Cooper et al., 2001). More recently, an increase of the ratio between the precursor of NGF, proNGF, and mature NGF, and imbalance in TrkA/p75^NTR^ ratio has been found in the brain and plasma from DS patients (Iulita et al., 2014; Iulita and Cuello, 2016; Miguel et al., 2021). This imbalance is known to trigger neurodegeneration (Capsoni and Cattaneo, 2006; Fahnestock and Shekari, 2019) and to contribute to neuroinflammation (Capsoni et al., 2011; Iulita et al., 2016). Indeed, similarly to AD, an activation of astrocytes and microglia, the main mediators of inflammation, has been reported in DS subjects (Wilcock and Griffin, 2013). Given these data, therapies aimed at re-establishing the correct balance between ligands of the NGF pathway appear to have a clear rationale (Cattaneo et al., 2008; Iulita and Cuello, 2014) also for DS.

**FIGURE 1:**
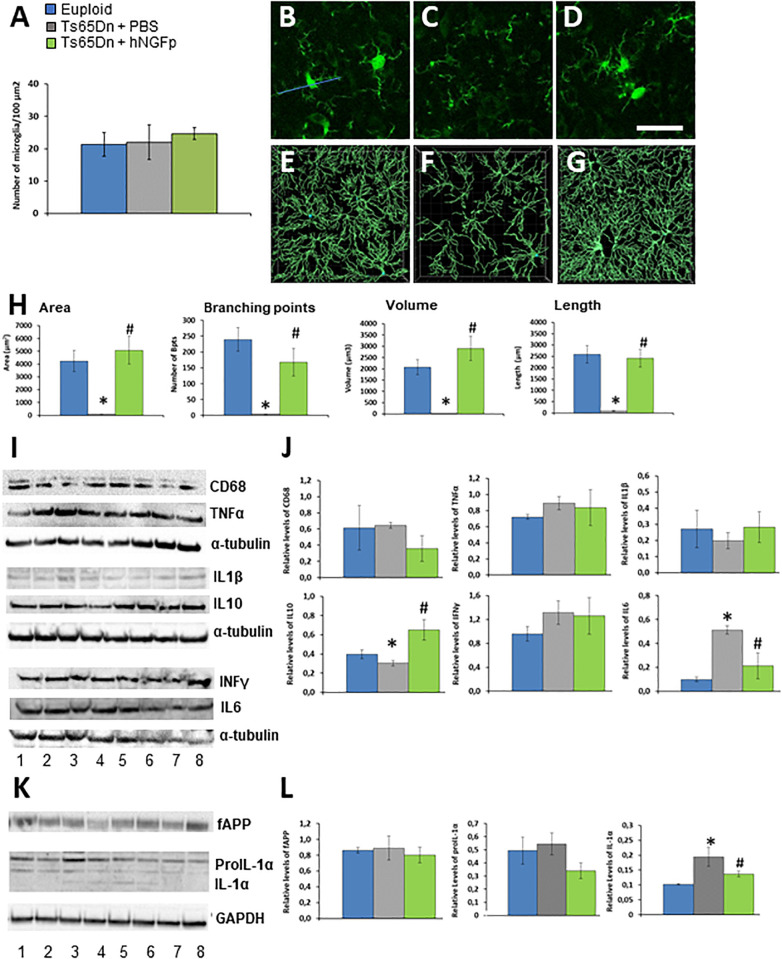
Intranasal hNGFp ameliorates microglial dystrophic morphology and reduces IL-1α levels in Ts65Dn mice. **(A)** Density of microglia cells in euploid, Ts65Dn mice treated with PBS or hNGFp. Immunohistochemistry for IBA-1 in cerebral cortex revealed morphological changes in panel **(C)** Ts65DN microglia with respect to panel **(B)** euploid mice. **(D)** hNGFp treatment rescues these morphological changes. Reconstruction of microglia cells by IMARIS: **(E)** euploid **(F)** Ts65Dn **(G)** NGFp-treated Ts65Dn mice. **(H)** Quantification of microglial morphological parameters. Bars are representative of mean ± SEM. *P < 0.001 vs. euploid mice, #*P* < 0.001 vs. Ts65Dn mice. *N* = 6/group. **(I)** Representative western blots and **(J)** densitometric analysis for CD68, TNFα, IL-1β, IL-10, and IL-6. **(K)** Representative western blots for APP and IL-1α species. Lanes 1–2 = euploid mice; 3–5 = Ts65Dn mice treated with PBS; 6–8 = Ts65Dn mice treated with hNGFp. **(L)** Densitometric analysis of APP, proIL-1α (graph on the left) and mature IL-1α (right panel) levels. Values have been normalized to GAPDH values. Bars are representative of mean ± SEM. *P < 0.001 vs. euploid mice, #*P* < 0.001 vs. Ts65Dn mice. *N* = 6/group. Scale bar = 10 μm.

We therefore tested the effect of intranasally delivered hNGFp in Ts65Dn mice at an early stage (4 months of age), prior to overt accumulation of APP and neurodegeneration [which in this model starts at 6 months of age (Choi et al., 2009; Figures 1K,L)]. We started by investigating whether morphological alterations in microglia are found at this early stage. Microglia was reported to be dystrophic in human DS brains (Streit et al., 2009), but was never studied in TS65Dn. By single cell morphologies from confocal images, we found that, despite a similar number of microglial cells (Figure 1A) in 4 months old Ts65Dn mice microglia (identified by Iba1 immunohistochemistry) is dystrophic, with a reduction in area, volume, length and number of ramifications (Figures 1C,F,H) with respect to euploid mice (Figures 1B,E,H). The intranasal administration of hNGFp significantly restored the morphology of microglia (Figures 1D,G,H). This is highly relevant, since the morphology of microglia is directly related to its functional state.

Then we measured the levels of markers of microglia activation and cytokines. As might be expected from the fact that we analyzed brains at an age in which Alzheimer-like neurodegeneration had not yet started, we did not find a differential expression among groups for CD68, TNFα, IL-1β, and INFγ (Figures 1I,J). On the contrary, we found that hNGFp increased the expression of IL-10 while decreasing IL-6 levels (Figures 1I,J). We found that IL-1α, which we know to be decreased in Alzheimer mouse models after hNGFp treatment (Capsoni et al., 2017), was decreased. IL-1α is produced as a precursor, proIL-1α, which is cleaved to an active, lower molecular weight molecule by calpain (Kobayashi et al., 1990; Carruth et al., 1991). We observed a reduction of proIL-1α in the brain extracts from Ts65Dn mice treated with hNGFp with respect to PBS-treated mice, although not statistically significant (Figures 1K,L, *P* > 0.05). On the contrary, a significant reduction of mature IL-1α (Figures 1K,L) was observed after hNGFp treatment, similarly to previous findings in hNGFp-treated 5xFAD mice (Capsoni et al., 2017).

**FIGURE 2:**
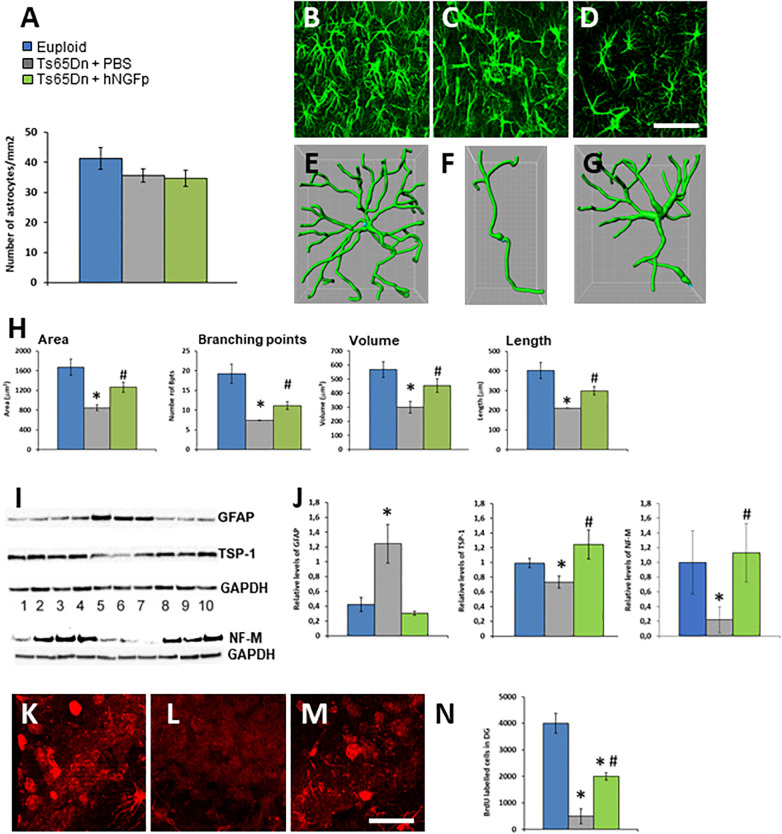
Intranasal hNGFp rescues astrogliopathy and neurogenesis deficit in Ts65DN mice. **(A)** Density of astrocytes in euploid, Ts65Dn mice treated with PBS or hNGFp. Immunohistochemistry for GFAP in hippocampus revealed morphological changes in panel **(C)** Ts65DN astrocytes with respect to panel **(B)** euploid mice. **(D)** hNGFp treatment rescues these morphological changes. Reconstruction of astrocytes by IMARIS: **(E)** euploid **(F)** Ts65Dn **(G)** NGFp-treated Ts65Dn mice. **(H)** Quantification of astrocytic morphological parameters. Bars are representative of mean ± SEM. *P < 0.05 vs. euploid mice, #*P* < 0.05 vs. Ts65Dn mice. *N* = 6/group. Scale bar = 35 μm. **(I)** Western blot for GFAP, TPS-1 and NF-M. Lanes 1–4 = euploid mice; 5–7 = Ts65Dn mice treated with PBS; 8–10 = Ts65Dn mice treated with hNGFp. **(J)** Densitometric analysis of GFAP, TPS-1, and NF-M levels. Values have been normalized to GAPDH values. Adult hippocampal neurogenesis is deficient in Ts65Dn mice compared to littermates and it is partially rescued by treatment with hNGFp. **(K–M)** examples from panel **(K)** euploid, **(L)** Ts65Dn, and **(M)** Ts65Dn dentate gyrus. **(N)** Stereological quantification of BrdU-labeled cells. Bars are representative of mean ± SEM. *P < 0.05 vs. euploid mice, #*P* < 0.05 vs. Ts65Dn mice. Scale bar = 200 μm.

Concerning astrocytes, we found no difference in their density among the treatment groups(Figure 2A). In PBS-treated 4 months old Ts65DN mice we found a significant reduction in volume, surface area, length and number of ramifications (Figures 2C,F,H) with respect to euploid mice (Figures 2B,E,H) in the hippocampus. These changes resemble the asthenic phenotype which precedes the astrogliosis observed in mouse models of AD and in early human AD (Verkhratsky et al., 2015). The intranasal delivery of hNGFp restores the characteristic shape of astrocytes in the brain of control mice, by increasing all parameters taken into consideration (Figures 2D,G,H). The characteristic shape of astrocytes is determined also by the expression the cytoskeletal protein GFAP. We found that, despite the reduction in volume, and consistently with what reported in literature for human DS (Jorgensen et al., 1990), there is an increase of GFAP levels in Ts65Dn mice compared to control mice (Figures 2I,J). This increase is completely reverted by the intranasal administration of hNGFp (Figures 2I,J).

In DS and in Ts65Dn mice cognitive deficits have been associated to structural abnormalities in dendritic spines. A critical factor for spine development is the production of thrombospondin 1 (TPS-1) by astrocytes. Indeed, decreased levels of TPS-1 have been found in the conditioned medium of cultured DS astrocytes and hypothesized to contribute to the reduced synaptogenesis (Torres et al., 2018). We found that also in the brain of 4 months old Ts65DN mice there is a decrease in TPS-1 protein, which was reverted to normal by the intranasal administration of hNGFp (Figures 2I,J).

Astrocytic homeostatic functions in the maintenance of neurogenesis are impaired in DS (Sloan and Barres, 2014). Moreover, we recently found that proNGF/NGF imbalance determines a reduced adult neurogenesis in the hippocampus dentate gyrus (Corvaglia et al., 2019). Also, adult neurogenesis in the dentate gyrus of young Ts65Dn mice showed markedly fewer BrdU-labeled cells than euploid animals (Clark et al., 2006). Based on these data, and on the fact that the intranasal administration of hNGFp increases the production of the chemokine CXCL12 (Capsoni et al., 2017) which is a pro-neurogenesis factor (Li et al., 2012), we measured the number of BrDU-immunoreactive cells in the dentate gyrus of 5 months old TS65Dn mice. We found a dramatic reduction of neurogenesis in the dentate gyrus of TS65Dn mice (Figures 2K–N), which was partially but significantly recovered by intranasal hNGFp (Figures 2K–N). Consistent with the increased neurogenesis with hNGFp, we found that hNGFp restored the levels the neuronal marker Neurofilament-M (Figures 2I,J), which was decreased in Ts65Dn mice.

In conclusion, we found that hNGFp treatment rescues astrogliosis, dystrophic microglia and neurogenesis deficits in the brain of 4 months old TS65Dn mice.

## General Conclusion and New Perspectives

From the data described in this perspective paper, we conclude that the links between deficits or alterations in the NGF system and AD go well beyond the long-established neurotrophic actions of NGF on BFCNs. Indeed, the intranasal delivery studies allowed to uncover that the cellular targets for NGF actions in the brain are more widespread than envisaged so far, including broadly distributed microglia and astrocytes. Given this finding, we conclude that intranasal hNGFp can be applied to other neurodegenerative and neurodevelopmental diseases in which cholinergic neurons are not the primary target of hNGFp action and neuroinflammation plays a relevant role. Thus, we conclude that the spectrum of neurodegenerative diseases that are amenable to be treated by hNGFp is very broad. In line with this conclusion, we presented new data demonstrating that intranasally delivered hNGFp has a potent neuroprotective action on the early phenotypic deficits in the TS65Dn mouse model of Down Syndrome and we propose that hNGFp could be used for the treatment of the progressive dementia affecting DS patients.

## Data Availability Statement

The original contributions presented in the study are included in the article/supplementary material, further inquiries can be directed to the corresponding author.

## Ethics Statement

The animal study was reviewed and approved by the Italian Ministry of Health.

## Author Contributions

SC designed the research, performed the research, and analyzed the data and wrote the manuscript. AC designed the research and wrote the manuscript. Both authors contributed to the article and approved the submitted version.

## Conflict of Interest

The authors declare that the research was conducted in the absence of any commercial or financial relationships that could be construed as a potential conflict of interest.

## Publisher’s Note

All claims expressed in this article are solely those of the authors and do not necessarily represent those of their affiliated organizations, or those of the publisher, the editors and the reviewers. Any product that may be evaluated in this article, or claim that may be made by its manufacturer, is not guaranteed or endorsed by the publisher.
